# Extra-neurohypophyseal axonal projections from individual vasopressin-containing magnocellular neurons in rat hypothalamus

**DOI:** 10.3389/fnana.2015.00130

**Published:** 2015-10-06

**Authors:** Vito S. Hernández, Erika Vázquez-Juárez, Mariana M. Márquez, Fernando Jáuregui-Huerta, Rafael A. Barrio, Limei Zhang

**Affiliations:** ^1^Departamento de Fisiología, Facultad de Medicina, Universidad Nacional Autónoma de MéxicoMexico City, Mexico; ^2^Departamento de Neurociencias, Centro Universitario de Ciencias de la Salud, Universidad de GuadalajaraGuadalajara, Mexico; ^3^Departamento de Física Química, Instituto de Física, Universidad Nacional Autónoma de MéxicoMexico City, Mexico

**Keywords:** paraventricular nucleus (PVN), juxtacellular labeling, vasopressin, extra-hypothalamic projections, axon collaterals, magnocellular neurosecretory system

## Abstract

Conventional neuroanatomical, immunohistochemical techniques, and electrophysiological recording, as well as *in vitro* labeling methods may fail to detect long range extra-neurohypophyseal-projecting axons from vasopressin (AVP)-containing magnocellular neurons (magnocells) in the hypothalamic paraventricular nucleus (PVN). Here, we used *in vivo* extracellular recording, juxtacellular labeling, *post-hoc* anatomo-immunohistochemical analysis and camera lucida reconstruction to address this question. We demonstrate that all well-labeled AVP immunopositive neurons inside the PVN possess main axons joining the tract of Greving and multi-axon-like processes, as well as axonal collaterals branching very near to the somata, which project to extra-neurohypophyseal regions. The detected regions in this study include the medial and lateral preoptical area, suprachiasmatic nucleus (SCN), lateral habenula (LHb), medial and central amygdala and the conducting systems, such as *stria medullaris*, the fornix and the internal capsule. Expression of vesicular glutamate transporter 2 was observed in axon-collaterals. These results, in congruency with several previous reports in the literature, provided unequivocal evidence that AVP magnocells have an uncommon feature of possessing multiple axon-like processes emanating from somata or proximal dendrites. Furthermore, the long-range non-neurohypophyseal projections are more common than an “occasional” phenomenon as previously thought.

## Introduction

According to the generally accepted theory of neurosecretion, arginine-vasopressin (AVP)-containing cells, located in the lateral division of the paraventricular nucleus of the hypothalamus (PVN), project to the neural lobe of the hypophysis via the paraventriculo-neurohypophysial tract. These neurons have large somata (diameters around 20–35 μm) and are traditionally referred to as magnocellular neurosecretory neurons (Armstrong, 2004), referred to as “magnocells” henceforward. The AVP-magnocells synthesize and release the nonapeptide vasopressin, which is critical for cardiovascular functions and hydro-electrolytic homeostasis (Bargmann and Scharrer, 1951).

It has been established that AVP-magnocell axons emanate either from the soma or a primary dendrite (Armstrong et al., 1980; van den Pol, 1982; Hatton et al., 1985; Rho and Swanson, 1989) and course in a wide arc, passing over or beneath the fornix before turning medially above the supraoptic nucleus to join the hypothalamo-neurohypophysial tract, reaching the internal medial eminence (MEI) and then the neural lobe. R. Greving was the first anatomist to describe this tract, coining the name *tractus paraventricularis-cinereus*, in a series of papers published in the early twentieth century (Greving, 1923, 1926, 1928). Hence, the tract also bears the name Tract of Greving. Axons from AVP magnocellular neurons have been seen to occasionally branch (van den Pol, 1982; Hatton et al., 1985; Ray and Choudhury, 1990), but the final destination and synaptic relationship of collaterals remain to be determined (Armstrong, 2004).

Besides the finding of dendritic AVP release in their vicinity, influencing local neuronal activity through paracrine mechanisms (Pow and Morris, 1989; Son et al., 2013), recent investigations using both *in vivo* and *in vitro* electrophysiological recording (Inyushkin et al., 2009), anatomical analysis and fluorogold retrograde tracing (Hernandez and Zhang, 2012; Zhang and Hernández, 2013) have suggested that the AVP-magnocells have important extra-neurohypophyseal axonal projections, establishing synaptic innervations in some limbic regions, such as the hippocampus (Zhang and Hernández, 2013) and the amygdala (Hernandez and Zhang, 2012). Activation of the AVP-magnocells modifies significantly the conditioned anxiety state (Zhang et al., 2012) and spatial learning (Hernandez et al., 2012).

Conventional neuroanatomical, immunohistochemical techniques, electrophysiological recording and *in vitro* labeling methods may fail to detect long-range extra-neurohypophysial-projecting axons of AVP-magnocells in the PVN. The difficulties include the large cell size for any kind of brain slide-based methods; the high somatic density inside the nucleus and the intermingled populations of both AVP parvo- and magno-cells; the chemical structural changes of the peptide from propressophysin to vasopressin during the intra-axonal transport (Brownstein et al., 1980), which decrease labeling with a single antibody (Zhang and Hernández, 2013); also the uneven axonal distribution of the dense core vesicles makes it confusing when following the thin axons under light and electron microscopy (Zhang and Hernández, 2013). In contrast, the *in vivo* extracellular recording with juxtacellular labeling technique (Deschênes et al., 1994; Pinault, 1994, 1996), combined with a *post-hoc* anatomo-immunohistological analysis, provides a powerful method to investigate the overall magnocellular neuron's morphology and its long-range projections.

Hence, the aim of this work is to reinforce our previous observation of the extra-neurohypophyseal AVP-containing innervation by AVP-magnocells, using *in vivo* juxtacellular single-neuron labeling, to accurately identify the extra-neurohypophyseal projections of these neurons from the PVN.

## Materials and methods

### Animals

Experiments were conducted in 155 adult male Wistar rats (250–400 g) provided by the local animal facility and housed at 20–24°C on a 12 h dark/light cycle (lights-on at 19:00 h) with tap water and standard rat chow pellets available *ad libitum*. The local bioethical and research committees approved all procedures involving experimental animals (approval ID CIEFM-086-2013).

### *In vivo* extracellular recording and juxtacellular labeling

The procedures for *in vivo* extracellular recording and juxtacellular labeling were based on the methods described in several references (Leng and Dyball, 1991; Pinault, 1996; Tukker et al., 2013). Anesthesia was induced with 4% isoflurane in oxygen, followed by urethane injection (1.3 g/kg, Sigma-Aldrich, intraperitoneal i.p.), with supplemental doses of xylazine (30 mg/kg), as necessary. Body temperature was maintained at 36°C with a heating pad. Once anesthetized, animals were placed on a stereotaxic frame and craniotomy was performed around the coordinates: −1.7 mm posterior to Bregma and 0.4 mm lateral. A long-taper glass electrode (8–15 MΩ) filled with 1–2% neurobiotin (Vector Laboratories) in 0.15 M NaCl was vertically placed at previously standardized hypothalamic PVN coordinates (Bregma: −1.7 mm; lateral: 0.4 mm; dorsoventral: 6.9 mm) and referenced against a wire implanted subcutaneously in the neck.

Extracellular single-unit activities under both basal conditions and hypertonic stimulation (NaCl 900 mM, 2% of body weight, i.p.) were detected, amplified and filtered using amplifiers ELC-01MX and DPA-2FL (npi electronics, GmbH, Tamm, Germany).

After recording, the cell was iontophoretically labeled with neurobiotin, using juxtacellular-labeling (Pinault, 1994). Current pulses of 1–10 nA, at 2.5 Hz, with a 50% duty cycle, were delivered through the recording electrode. The current was gradually increased to induce and maintain entrainment of the activity of the neuron, yielding a higher number of spikes on the current “on” periods. Cells were entrained for 1–10 min.

### Tissue processing and immunohistological analysis

Two to five hours after labeling a cell, cardiac perfusion with saline was performed and followed by 15 min of fixation with a solution containing 4% paraformaldehyde, 15% (v/v) saturated picric acid and 0.05% glutaraldehyde in 0.1 M phosphate buffer (PB). Each recorded cell was coded with the electrophysiologist's initials followed by the sequential number of experiments for internal control.

Coronal sections cut at 70 μm thickness with a vibratome (Leica VT 1000S) were stored serially in tissue culture wells in 0.1 M PB containing 0.05% NaN_3_. Four to six sections containing the electrode tract were initially incubated with streptavidin-conjugated Alexa Fluor 488 (1:1000; Invitrogen) in TBST (Tris-buffered saline with 0.3% Triton X-100) for 1 h at room temperature (RT). Sections mounted in VectaShield (Vector Laboratories, Burlingame, CA) were assessed under light microscope. Sections of interest (containing well-labeled soma or axon/axon collaterals) were de-mounted and further incubated, first with 10% normal donkey serum in TBST and with rabbit anti-AVP (1:2000; Peninsula Laboratories, T-4563), the specificity of which was confirmed by the absence of immunoreactivity after preabsorption with vasopressin peptide (Taylor et al., 2008), and/or guinea-pig anti-vesicular glutamate transporter (vGluT2-GP-Af810, 1:1000, Frontier Institute Co., LTD, Ishikari, Japan) the specificity of which was confirmed via immunoblotting and preabsorption (Miyazaki et al., 2003).

### Camera lucida reconstruction of AVP+ magnocells labeled with neurobiotin

Six neurobiotin well-labeled AVP+ magnocells were selected for reconstruction, described elsewhere (Losonczy et al., 2002; Tukker et al., 2013). Briefly, immunohistochemically processed sections were de-mounted again and converted to polymerized 3,3' diaminobenzidine (DAB) horseradish peroxidase (HRP) end product for analysis of neuronal processes at high resolution. This was done by incubating first the sections containing streptavidin-fluorescence signals with component B (1:100) of Vector ABC Elite kit overnight and then incubating them with A+B (1:100 each, in 0.1 M PB, Vector ABC Elite kit) for 1 h at room temperature. Reactions for HRP were done with DAB (0.05%) as a chromogen and 0.01% H_2_O_2_ as a substrate. Sections were air-dried and mounted with Permount Mounting Medium (Electron Microscopy Sciences, Hatfield, PA). Further adjacent sections were chosen in an alternate manner to process with streptavidin-conjugated Alexa Fluor 488 (1:1000; Invitrogen) to establish the extension of neurobiotin labeled processes. Once the labeling borders were defined, these sections were processed as described above. The remaining sections inside the defined limits were converted to DAB-HRP end product using freeze-thaw permeabilization (no detergent used). Neurobiotin was revealed by the glucose oxidase method (Shu et al., 1988), further treated with OsO_4_ (0.1–1%) and mounted on slides in epoxy resin for future electron microscopy study.

The somatic, dendritic and axonal patterns of each neuron were analyzed at high magnifications and were subsequently reconstructed using a drawing tube (Nikon; for methodological references see Losonczy et al., 2002). The soma and dendrites were represented in black and axonal segments were represented in red. Some short finely beaded process of “unknown nature” as defined by Sofroniew and Glasmann (1981), were labeled in green. The “main axons” were judged by the classical description of beaded neurosecretory axons, which emit laterally from either the soma or a primary dendrite and curse in a wide arc, passing over or beneath the fornix before turning medially above the supraoptic nucleus and optical tract, toward the MEI (Armstrong, 2004). The “axon-collaterals” were assigned to the branches arisen from the main axons and the “axon-like” processes were judged by either expressing vesicular glutamate transporter 2 (vGluT2) and/or being long distance projection and/or branched extensively (axon terminals) with constantly thin “strings of pearls” shaped swellings (Sofroniew, 1983; Armstrong, 2004).

For a clearer visualization from a different angle of the labeled AVP immunopositive neuron EV16, we also performed computer aided reconstructions based on the light microscopic observations and Brain Atlas (Paxinos and Watson, 2006) using a generic 3D content creation suite BLENDER (http://www.blender.org/).

### Identification criteria for paraventricular vasopressin containing magnocellular neuroendocrine neurons (AVP-magnocells)

We proceeded with juxtacellular labeling when the recorded cells showed a slow and irregular firing pattern under basal condition and increased firing rate under hypertonic stimulus (Gainer, 1972; Leng and Dyball, 1991). Individually labeled somata located in the lateral paraventricular region complying the anatomical criteria established in the literature (Harris, 1948; Bisset et al., 1967; Hayward and Jennings, 1973; Sofroniew, 1983; Leng and Dyball, 1991; Armstrong, 2004), exhibiting a long, thick and beaded process joining the tract of Greving, were selected to test immunoreactivity to AVP. The positive cases were further filtered with the criterion of labeling span, i.e., only the cells having a labeling span of more than 20 sections, 1.4 mm in antero-posterior axis, were considered well labeled.

## Results

**Figure 1:**
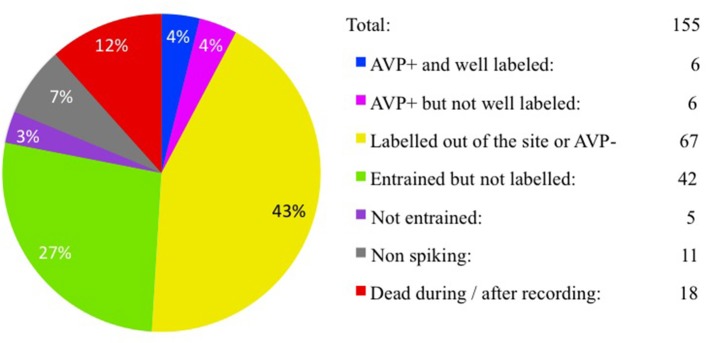
**Juxtacellular recording/labeling census (cell per rat)**. Pie-chart showing recording/labeling census of this study. One hundred fifty-five rats were used for *in vivo* extracellular recording and juxtacellular labeling of hypothalamic paraventricular vasopressin-containing magnocellular neurons (AVP-magnocells). The fraction of cases that satisfied the selection criteria is labeled in blue.

We recorded the activity of vasopressin containing magnocells in the hypothalamic PVN in anesthetized rats with extracellular glass electrodes. After recording the physiological activity, the recorded cells were approached by the electrode and filled with neurobiotin using juxtacellular-labeling technique. From 155 recordings performed, six cells from six rats were found to satisfy the selection criteria. The cell bodies were in the PVN, lateral magnocellular division (PaLM, *n* = 3, EV16, MM22, and VH25) or posterior division (PaPo, *n* = 3, EV40, VH52, and MM15). All six cells were tested to be immunopositive for AVP and had more than 20 s (70 μm of thickness each) with labeled soma and/or processes, as well as exhibiting characteristic electrophysiological properties during *in vivo* recording. A census of all recordings and labeling for this study is given in Figure 1.

### Morphological features of AVP-magnocells recorded in the PaLM

**Figure 2:**
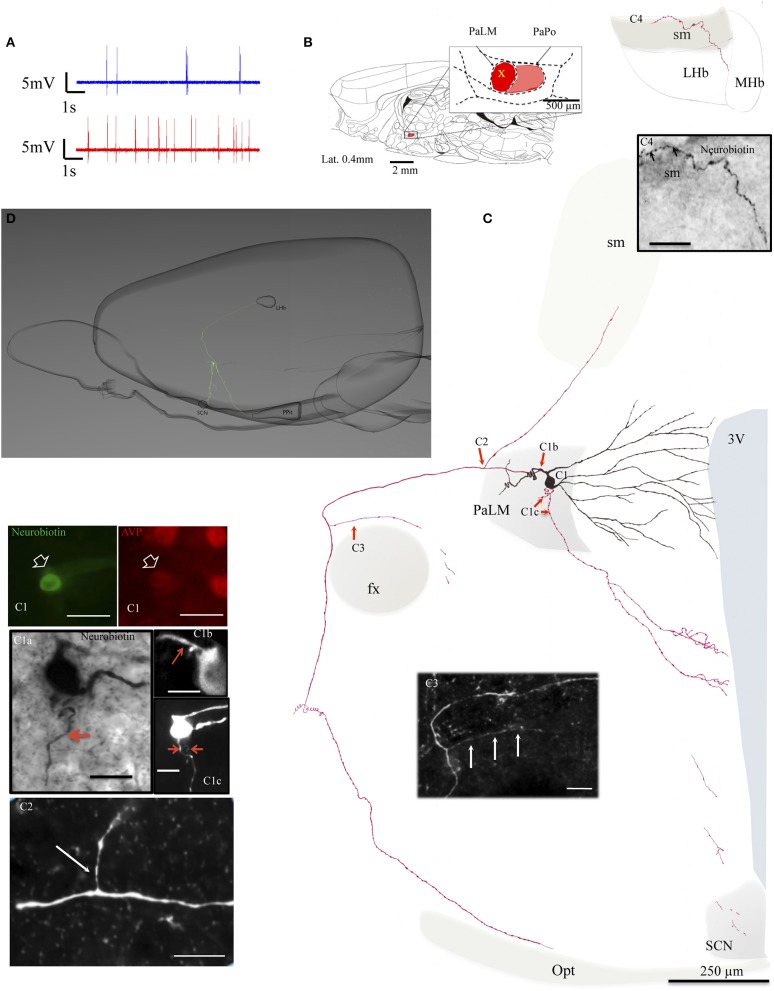
**Neuron EV16**. **(A)** Spike trains under basal (blue trace) and hypertonic (red trace) conditions. **(B)** Sagittal plate of lateral 0.40 mm from Rat Brain Atlas (Paxinos and Watson, 2006) and the amplification of the paraventricular nucleus (inset): red and pink shadows indicate the lateral magnocellular division of paraventricular nucleus (PaLM) and the posterior division (PaPo) respectively. The yellow cross indicates the rostro-caudal position of EV16. **(C)** Camera lucida reconstruction of the neuron's soma, dendrites and axons. Drawings from serial sections were superimposed manually in a 2-dimension (2D) projection drawing on a coronal view of the rat hypothalamus. The soma and dendrites were represented in black and axonal segments were represented in red. **(C1)** AVP-containing nature was ascertained by AVP immunoreaction. The soma gave rise initially to two short thick primary dendrites, which branched proximally. The bottom dendrite branched extensively until the fifth order of branches; all directed medially reaching the wall of the third ventricle (3V). The top dendrite gave rise to two secondary branches, the medial and the lateral ones. The medial branch was similar to the bottom group. The lateral portion curled up proximally near the soma but gave rise to the main axon. Photomicrograph (**C1a**, from neurobiotin HRP/DAB reaction); (**C1b,C1c**, from neurobiotin-streptavidin-Alexa 488 fluorescence in two consecutive sections) showing the sites where three axons were originated (red arrows). The main axon coursed laterally passing the fornix (fx), turned ventro-caudally toward the posterior pituitary gland (PPit) **(D)**. Two main collaterals emanated from this axon **(C2,C3)**. The first collateral **(C2)** coursed dorsomedially joining the *stria medularis* (sm). Neurobiotin labeled processes were found inside the lateral habenula **(C4)**. The second collateral originated passing the fx and coursed medially **(C3)**. The labeling of one of the ventrally directed axons coursed further ventrally along the periventricular region, reaching the suprachiasmatic nucleus (SCN). **(D)** Computer aided schematic reconstruction based on observations of serial coronal sections to facilitate the perspective of axonal projections from a sagittal view. Note that the size of cell vs. brain is not in strict proportion. LHb and MHb, lateral and medial habenula; PPit, posterior pituitary gland; sm, *stria medularis*; fx, fornix; 3V, third ventricle; opt, optical tract. Scale bars: 50 μm for **(C1)**, 20 μm for the rest of panels.

The location of cell bodies was ascertained on the basis of neurobiotin—streptavidin—Alexa 488 reaction on serial sections in/around the electrode penetration site. The dendrites and axons in the consecutive sections were drawn under 100× objective with the help of a drawing tube and superimposed manually in a 2-dimension (2D) projection drawing on a coronal view of the rat hypothalamus, except for EV16, in which a 3D computer aided reconstruction for a lateral view was also made (Figure 2D). The neuron EV16 fired slow irregular spike trains under basal conditions (< 1 spike/s) but increased its firing rate measured 5 min after the i.p. hypertonic saline injection (1.8 spikes/s) (Figure 2A, blue and red traces, respectively). The soma of EV16 was located in the medial part of PaLM (Figure 2B), with its long axis 30° oblique to the midline (Figure 2C). The soma gave rise initially to two short and thick primary dendrites, which branched proximally. The bottom dendrite branched extensively until the fifth order of branches—all directed medially reaching the wall of the third ventricle (3V). The top dendrite emitted two secondary branches, the medial and the lateral ones. The medial branch was similar to the bottom group. The lateral branch curled up proximally near the soma but gave rise to the main axon. The main axon coursed laterally passing on top of the fornix (fx), turned ventrally and then medio-posteriorly (Figures 2C,D). Further caudal labeling could not be established due to high background DAB reaction and ruptures due to freeze-thaw permeabilization procedures. Two main collaterals were observed. C2 indicates the first branch position. This tracer containing process coursed dorsally and reached the border of the *stria medullaris* (sm). The continuation was lost temporally in the myelinated conducting structure but was established again in the surface of sm and entered the lateral habenula (LHb) (Figure 2C4). The second branch arose from the parent axon (Figure 2C3), coursed medially and was lost in the border of the fornix. One of the ventrally directed axons coursed further ventrally along the periventricular region, reaching the suprachiasmatic nucleus (SCN), where labeled axonal processes were found. Another labeled axon process ended inside the PaLM (Figure 2C1c).

**Figure 3:**
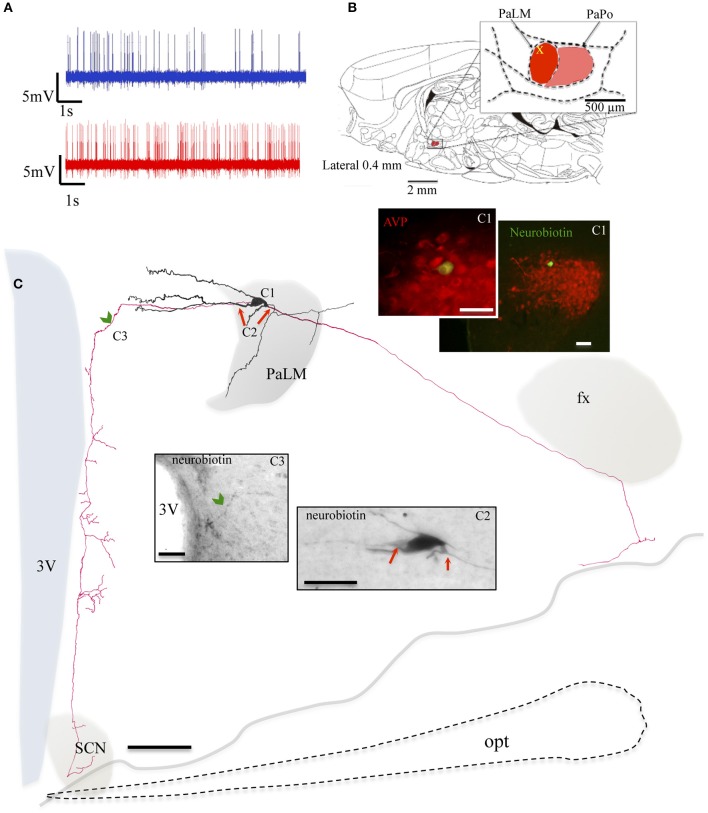
**Neuron MM22**. **(A)** Spike trains under basal (blue trace) and hypertonic (red trace) conditions. **(B)** Sagittal plate of lateral 0.40 mm from Rat Brain Atlas (Paxinos and Watson, 2006) and the amplification of the paraventricular nucleus (inset): red and pink shadows indicate the lateral magnocellular division of paraventricular nucleus (PaLM) and the posterior division (PaPo) respectively. The yellow cross indicates the rostro-caudal position of MM22. **(C)** Camera lucida reconstruction of the neuron's soma, dendrites and axons. Drawings from serial sections were superimposed manually in a 2-dimension (2D) projection drawing from a coronal view of the rat hypothalamus. The soma and dendrites were represented in black and axonal segments were represented in red. AVP containing nature of MM22 was ascertained by AVP immunoreaction **(C1)**. Photomicrographs from neurobiotin HRP/DAB reaction in **(C2,C3)** show the sites where two main axons were originated (red arrows) and where the axon turned medially (green arrowhead) and coursed ventrally along the periventricular zone emitting numerous branches toward the suprachiasmatic nucleus (SCN). opt: optical tract, fx: fornix. The light gray irregular line symbolizes the site where the brain tissue was broken. The optical tract, symbolized by dashed line was drawn only for reference. Scale bars: 100 μm in main drawing, 50 μm in **(C1)**, and 30 μm in **(C2,C3)**.

**Figure 4:**
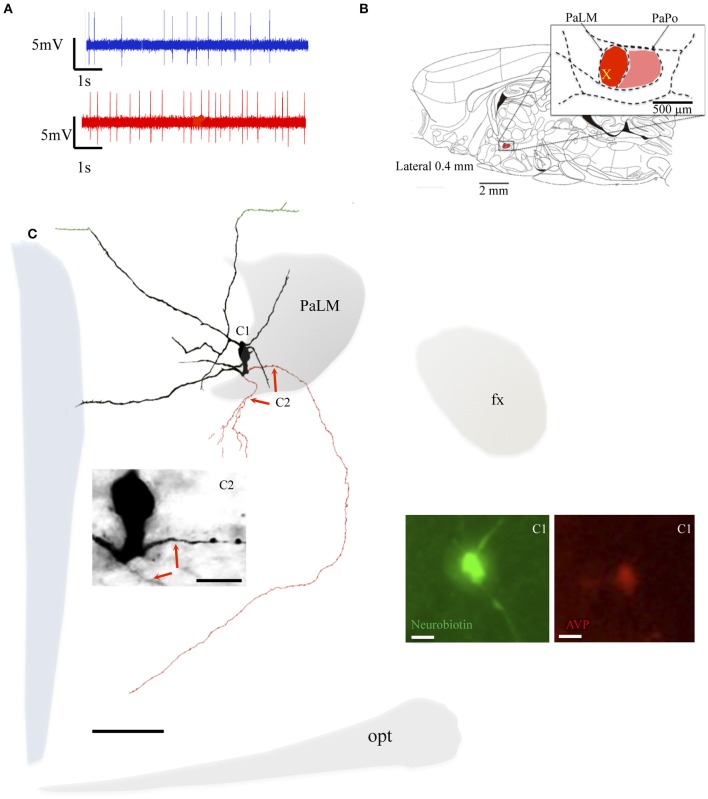
**Neuron VH25**. **(A)** Spike trains under basal (blue trace) and hypertonic (red trace) conditions. **(B)** Sagittal plate of lateral 0.40 mm from Rat Brain Atlas (Paxinos and Watson, 2006) and the amplification of the paraventricular nucleus (inset): red and pink shadows indicate the lateral magnocellular division of paraventricular nucleus (PaLM) and the posterior division (PaPo) respectively. The yellow cross indicates the rostro-caudal position of VH25. **(C)** Camera lucida reconstruction of the neuron's soma, dendrites and axons. Drawings from serial sections were superimposed manually in a 2-dimension (2D) projection drawing on a coronal view of the rat hypothalamus. The soma and dendrites were represented in black and axonal segments were represented in red. The green processes indicate the beaded processes originated from dendritic processes. This kind of processes were described in an early study and were considered as axonal processes (Sofroniew and Glasmann, 1981). Numbered regions/segments in **(C)** correspond to the numbered photomicrographs. **(C1)** Vasopressin (AVP)-containing nature of neuron VH25 was ascertained by AVP immunoreaction; **(C2)** photomicrograph from neurobiotin HRP/DAB reaction showing the sites where the two main axons were originated (red arrows) opt, optical tract; fx, fornix. Scale bars: 100 μm for main panel, 25 μm for **(C1,C2)**.

During the *in vivo* recording, the neuron MM22 fired irregularly under basal conditions and showed a firing rate of 4.5 spikes/s (Figure 3A, blue trace). Five minutes after the i.p. hypertonic saline injection, the cell fired with a frequency of 10.2 spikes/s (Figure 3A, red trace). The soma was located in the medial part of the PaLM mid-way through its anterio-posterior axis (Figure 3B) and emanated three primary dendrites with few further branches (Figure 3C). Similar to EV16, the dendrites were mainly orientated toward the medial direction. Two main axons were found originating from the soma and a proximal dendrite, (Figure 3C2). The axon that coursed laterally joined the tract of Greving. The labeling was lost when it turned medially due to the loss of tissue during the slicing procedure. The medial axon turned ventrally near the 3V (Figure 3C3) and coursed along the periventricular region emitting numerous axon branches. The longest distance of branches was found in the dorsal part of the SCN. Neuron VH25 (Figure 4) is another *in vivo* recorded and labeled AVP-magnocell with similar physiological properties and somatic, dendritic and axonal distributions.

### Morphological features of AVP-magnocells recorded in paraventricular nucleus posterior part (PaPo)

**Figure 5:**
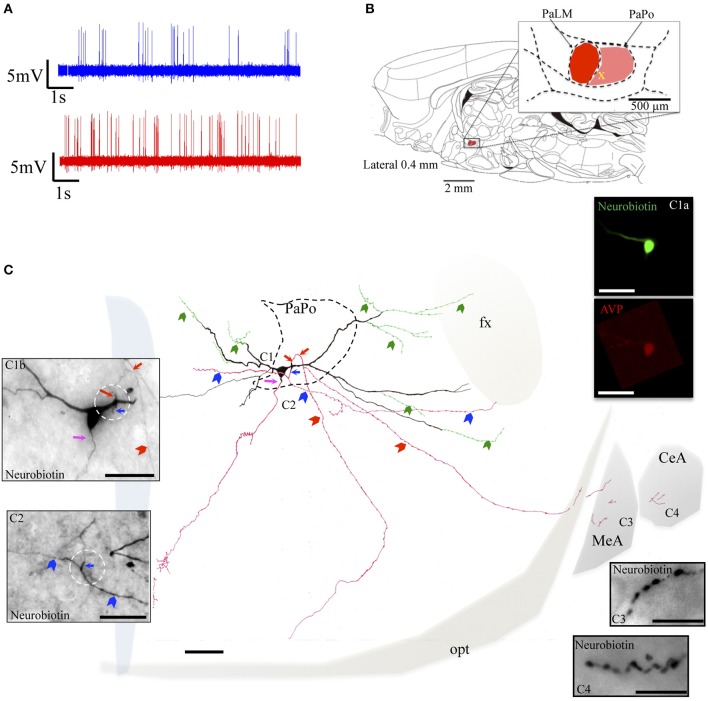
**Neuron EV40**. **(A)** Spike trains under basal (blue trace) and hypertonic (red trace) conditions. **(B)** Sagittal plate of lateral 0.40 mm from Rat Brain Atlas (Paxinos and Watson, 2006) and the amplification of the paraventricular nucleus (inset): red and pink shadows indicate the lateral magnocellular division of paraventricular nucleus (PaLM) and the posterior division (PaPo) respectively. The yellow cross indicates the rostro-caudal position of EV40. **(C)** Camera lucida reconstruction of the coronal projection and corresponding photomicrographs **(C1–C4)** showing dendritic and axonal patterns. Red, blue and pink arrows indicate the origins of three parent-axons. Red and blue arrowheads indicate the subsequent axonal branches. The green arrowheads indicate the beaded processes originated from the dendritic processes, some of them entering the fornix (green arrowheads). This kind of processes were described in an early study and were considered as axonal processes (Sofroniew and Glasmann, 1981). **(C1a)** AVP-containing nature was ascertained by AVP immunoreaction; **(C2)** photomicrograph of the section contiguous caudally to the soma (depicted in **C1b**) showing the branching of the axon labeled with a blue arrow. **(C3,C4)** Show axonal processes found in amygdala. Scale bars: 100 μm for **(C)**; 50 μm for all photomicrographs. MeA, medial amygdala; CeA, central amygdala; opt, optical tract; fx, fornix.

The selected neurons EV40, VH52, and MM15 were located in the PaPo, which was not our original labeling target. The unexpected labeling was probably due to the Bregma variation among rats and the narrow antero-posterior extension of the PaLM (about 200 μm). However, the vasopressin-containing neurons recorded and labeled in these regions fulfilled the selection criteria. The neuron EV40, located in the ventral part of the PaPo (Figure 5B), fired spontaneous action potentials *in vivo* and increased its firing rate after hypertonic saline injection (2.5 spikes/s vs. 5.9 spikes/s, Figure 5A). Unlike the three neurons inside the PaLM, this neuron had its dendritic arborization extended both medially and laterally. Anatomical analysis showed that this neuron clearly possessed 3 axons. One dendritic point proximal to the soma gave origin to two primary (parents) axons in opposite directions (Figure 5C, red and blue arrows, both in the drawing and the photomicrographs Figures 5C1b,C2, white circled region). The dorsal primary axon turned ventrally to further branch into two secondary axons, one projected to the tract of Greving and the other projected to the border of the optical tract (red arrowheads). Labeled axon traces were found inside the medial and central amygdala, which should be the extensions of this axon (some tissue was lost during its processing). The ventral primary axon (blue arrow) was split into two branches of axon-collaterals (indicated with blue arrowheads) directed either medially or laterally. The lateral branch entered the fornix (fx). A third axon left the soma at the site indicated by the pink arrow and coursed medio-ventrally reaching the ventral periventricular region. Additionally, several beaded processes (green arrowheads) were originated from dendrites, some of them entered the fornix. This kind of processes was described in an early study and were considered to be axonal processes (Sofroniew and Glasmann, 1981).

**Figure 6:**
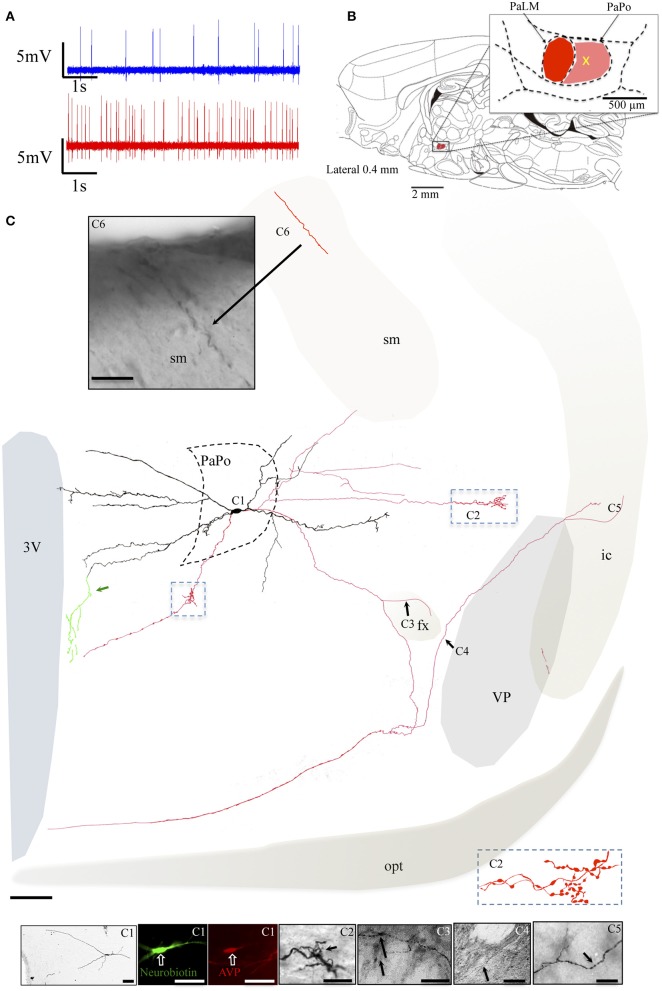
**Neuron VH52**. **(A)** Spike trains under basal (blue trace) and hypertonic (red trace) conditions. **(B)** Sagittal plate of lateral 0.40 mm from Rat Brain Atlas (Paxinos and Watson, 2006) and the amplification of the paraventricular nucleus (inset): red and pink shadows indicate the lateral magnocellular division of paraventricular nucleus (PaLM) and the posterior division (PaPo), respectively. The yellow cross indicates the rostro-caudal position of VH52. **(C)** Coronal projection of camera lucida two-dimensional reconstruction and corresponding photomicrographs **(C1–C6)** showing dendritic and axonal patterns. **(C1)** Shows that the AVP-containing nature was ascertained by AVP immunoreaction; **(C2)** shows an axon with several small branches and classical image of strings of pearls in a small region in the lateral preoptical area; **(C3–C6)** show the presence of labeled axon segments in or near the conducting systems, fornix (fx), internal capsule (ic), and *stria medullaris* (sm). Green traces indicated by the green arrow, showing the beaded processes originated from the dendritic processes branching extensively in the periventricular region. This kind of processes were described in an early study and were considered as axonal processes (Sofroniew and Glasmann, 1981). Scale bars: 100 μm for **(C)**; 50 μm for all photomicrographs. VP, Ventral pallidum (symbolized with gray shadow); conducting systems (i.e., optical tract, opt, *stria medullaris* st, fornix, fx, internal capsule, ic) were symbolized with light beige shadows.

**Figure 7:**
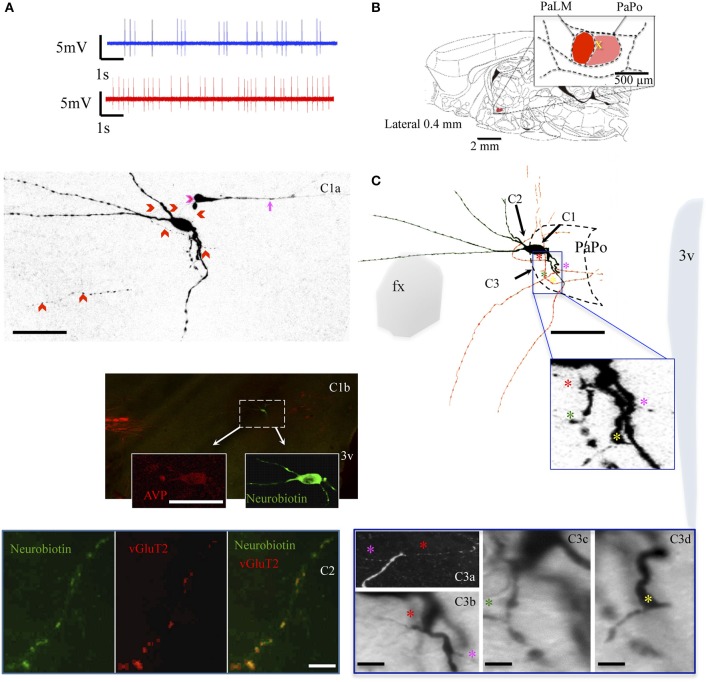
**Neuron MM15**. **(A)** Spike trains under basal (blue trace) and hypertonic (red trace) conditions. **(B)** Sagittal plate of lateral 0.40 mm from Rat Brain Atlas (Paxinos and Watson, 2006) and the amplification of the paraventricular nucleus (inset): red and pink shadows indicate the lateral magnocellular division of paraventricular nucleus (PaLM) and the posterior division (PaPo) respectively. The yellow cross indicates the rostro-caudal position of MM15. **(C)** Camera lucida reconstruction of coronal projection and corresponding photomicrographs **(C1–C3)** showing dendritic and axonal patterns. The soma was located in the PaPo anterolateral border with PaLM. It emitted three primary dendrites. In contrast to the previous cells, this cell's main dendritic arborizations were directed laterally and ventrally. Four axonal processes (colored asterisks) arose from the proximal dendritic loci belonging to the same primary dendrite. Two of them projected ventrally joining the tract of Greving. The two other axon-like processes bended dorsally. **(C1a)** Shows a projection of several confocal photomicrographs of the juxtacellular labeled cell. Note that there was a process labeled from an unlabeled soma, indicated by a pink arrow. It seemed that the tracer passed from MM15 to this segment through the contact indicated by a pink arrowhead, also see the Supplementary Video. This phenomenon has been reported in the literature and the presence of gap-junctions between the two structures had been suggested (Andrew et al., 1981), **(C1b)** and insets show the AVP-containing nature of the labeled cell ascertained by AVP immunoreaction. **(C2)** Shows a thin axon-like process positive to vGluT2. **(C3a)** Is a digital rotation of the 3D reconstruction (Supplementary information) aiming to show the double axons (labeled with red and pink asterisk) emitting from the same point of the proximal dendrite (**C3b** is a coronal view of the same feature). **(C3c,d)** Show the emerging sites of the two other axons. Scale bars: for **(C,C1a,b)** 50 μm; rest: 10 μm.

The neuron VH52 had similar electrophysiological behaviors as EV40 during *in vivo* recording (Figure 6A). The neuron fired irregular spontaneous action potentials *in vivo* and increased its firing rate after hypertonic saline injection (1.2 spikes/s vs. 4.8 spikes/s, Figure 6A). The soma was located in the medial part of the PaPo (Figure 6B). The dendritic and axonal extensions are depicted in Figure 6C. Vasopressin (AVP)-containing nature was ascertained by AVP immunoreaction (Figure 6C1). A particular structure was observed: axon with several small branches and classical image of *strings-of-pearls* in a small region in both medial preoptical region and lateral hypothalamic area (Figure 6C, blue square and Figure 6C2). The main axon coursed ventral-laterally joining the tract of Greving. This axon emitted a collateral entering the fornix (fx) (Figure 6C3). The main axon coursed further and curled and branched at the basal forebrain region. One branch coursed dorso-laterally entering the ventral pallidum (VP) and internal capsule (ic). Unfortunately, one part of the tissue was lost during the slicing due to insufficient fixation (Figure 6C, irregular light gray line depicts the fracture). In Figures 6C3–6 show the presence of labeled axon segments in or near the conducting systems, fornix (fx), internal capsule (ic), and *stria medullaris* (sm). The cell MM15 also increased its firing rate after hypertonic saline injection (1.9 spikes/s vs. 3.2 spikes/s, Figure 7A). It was located in the top lateral border of PaPo (Figure 7B). In contrast with EV16, MM22 and VH25, which were located in the medial part of the PVN, this neuron's dendrites extended laterally. The medial proximal dendrites gave origin to two tract of Greving-projecting thick varicose axons and two locally branching thin axons, which tested to be vGluT2 immunopositive (Figure 7C and Supplemental information).

## Discussion

We have used *in vivo* electrophysiological recording and juxtacellular labeling procedures for *post-hoc* anatomical visualization of single vasopressin containing magnocellular neurons in the hypothalamic PVN of anesthetized rats. Our results revealed some unusual and important anatomical features at single cell level: (1) all six cells that met the inclusion criteria for vasopressin-containing magnocells possessed multi-axons originated in the soma or a proximal dendrite and four out of the six cells also exhibited axon collaterals branching from the main axons; (2) in addition to the axons, which joined the tract of Greving, all these cells projected also to other intra- and extra-hypothalamic regions; (3) some of the downstream regions were identified by localizing labeled axon/axon terminals.

### New contributions to the morphology and efferent paths of paraventricular AVP containing magnocells

In the late 70s, R. M. Buijs and colleagues provided detailed characterization of intra- and extra-hypothalamic innervation of vasopressin containing fibers, specifically the connections with limbic regions (Buijs, 1978; Buijs and Swaab, 1979). However, the knowledge on the origin of these AVP innervations is limited. There have been some excellent studies on the morphology of the magnocellular neurosecretory neurons (magnocells) using immunohistochemical and Golgi-like immunoperoxidase staining methods (Sofroniew and Glasmann, 1981; van den Pol, 1982; Armstrong, 2004). However, the quasi-two-dimension nature of the previous methods (i.e., labeling and reactions on brain slices, as thick as 350 μm) impaired crucially the overall visualization of individual magnocellular neurons. This is particularly true regarding the efferent pathways. Traditionally it has been described that the axons of paraventricular magnocells egress laterally from either the soma or a primary dendrite (Armstrong et al., 1980; van den Pol, 1982; Hatton et al., 1985; Rho and Swanson, 1989) to join the tract of Greving before reaching the medial eminence internal part and the neural lobe (Armstrong, 2004). It was thought that the PVN AVP+ axons from magnocellular neurons only occasionally branch (van den Pol, 1982; Hatton et al., 1985; Ray and Choudhury, 1990).

*In vivo* extracellular recording and juxtacellular neuronal labeling technique has proven to be a powerful method to decipher the morpho-functional relationship at a single neuron level and its projection targets (Pinault, 1996). It provides precise information of anatomical location, morphology and neurochemical phenotype, of the neuron labeled (Toney and Daws, 2006) and long range axon projections. Herein, we report that all the AVP-containing magnocellular neurons that met the inclusion criteria we stated in the method section for this study, possessed multi-axonal projections or axon collaterals branching very near the soma projecting to several brain regions, such as the preoptical and anterior hypothalamic areas and SCN; it is especially interesting to see a long range projection which was detected entering the *stria medularis* fiber-system innervating LHb (Figures 2C,D).

The labeled neurons reported in this study were grouped into two classes. The first three cells, EV16, MM22, and VH25 were located in the medial part of the PaLM region. These neurons showed some similarities in their dendritic and axonal branching patterns: their dendrites predominantly extended toward the medial parvocellular region of the PVN, reaching the border of the third ventricle (3V) and all the three neurons possessed at least two axons, the lateral-bound joining the tract of Greving and the medial-bound axon. The region between the PaLM and the 3V consists of the main population of corticotrophin releasing factor (CRF) synthesizing neurons, which mainly project to the medial eminence portal system to regulate the stress response. This dendritic distribution at single cellular level, adding to the evidence that CRF containing axon terminals establish Gray type I synapse with AVP+ dendritic segments in this region (Zhang et al., 2010), and that AVP dendritic release in their vicinity influence the local activity through paracrine mechanisms (Pow and Morris, 1989; Morris et al., 1998; Son et al., 2013), suggests an important crosstalk between these two neuronal populations, especially under stressful conditions, to coordinate body's adaptive responses. Since 1934, M. I. Laruelle described two groups of paraventriculo-hypophyseal fibers, an external and an internal set (Harris, 1948), which can be observed clearly under conventional immunohistochemical reaction against AVP. Our contribution to this established concept is that, at least some of these AVP+ fibers have the same cellular origin as the lateral-bound ones. The structure-functional relationship for this arrangement remains to be established. The demonstration that axons projecting to the tract of Greving also emitted two axon collaterals to the lateral hypothalamus and to the LHb, through *stria medullaris*, is of specific interest. Recently, we have observed that the AVP-containing axons establish synaptic innervation onto LHb neurons and its up-regulation promotes motivational behavior during multifaceted stress coping by suppressing the functional activation of this region (unpublished data). Hence, this observation provides one cellular substrate to understand this neural circuit function.

The neuron MM15 was found located on the postero-lateral border between PaLM and PaPo. In contrast with the neurons previously mentioned, this neuron extended its dendrites mainly to the lateral hypothalamus. It seemed that the magnocells tend to extend their dendrites avoiding the densely packed PaLM regions. Afferents for these dendritic segments remain to be determined. This cell emitted several shortly branching axons.

The two neurons located in the PaPo regions (EV40 and VH52) had symmetric patterns regarding their dendritic arborization. Both neurons exhibited clear multi-axonal features sending projections to the conducting systems such as fornix, optical tract, and internal capsule, also to the medial and central amygdala. Several other reports found in the literature are in good agreement with our observations (Sofroniew and Glasmann, 1981; Inyushkin et al., 2009). In spite of their location in PaPo, these two neurons had similar physiological behavior to the previous cells located inside the PaLM region, during the osmotic challenge. We have observed Fos expression in this region, post-salt loading (unpublished data), which is in congruence with the observation from this study.

It is generally considered that the magnocellular neurosecretory neurons of PVN and SON, almost exclusively, project through the *zona interna* of the median eminence to the posterior pituitary, where they terminate at fenestrated capillaries and secrete AVP and OT into the blood circulation upon appropriate stimulation (Armstrong, 2004). Sofroniew and Glasmann by using a Golgi-like immunoperoxidase staining, first reported three types of processes originating from the soma of AVP immunoreactive magnocells (Sofroniew and Glasmann, 1981; Sofroniew, 1983): (1) beaded processes; (2) dendritic processes, and (3) thick peptide containing processes. The first group can be originated from the perikaryon, a dendrite or the main axon (as axon collaterals). They had an appearance similar to the classical descriptions of beaded neurosecretory axons, hence were considered by the authors as axons (Sofroniew and Glasmann, 1981). However, this observation had not received widespread appreciation due to the possible ambiguity of the method. Inyushkin et al. using *in vivo* electrophysiological recording, demonstrated a clear dual projection system, neurohypophyseal and central, from the AVP-immuno-positive magnocellular neurons of the SON and suggested the existence of projections from these neurons in the SON that are much more widespread and longer than had previously been suspected (Inyushkin et al., 2009). The observations obtained from this study complement the previous observation providing additional anatomical evidence of the multi-axonal nature of the hypothalamic magnocellular neurosecretory neurons.

It is now known that hypothalamic neurosecretory neurons not only control the body homeostasis but also exert powerful control on individual emotional states, social behavior and decision making (Stoop, 2012). Our group has published a series of papers in recent years on the AVP magnocell's influence on hippocampal function and anxiety (Hernandez et al., 2012; Zhang et al., 2012; Zhang and Hernández, 2013). Fluorogold retrograde tracing from hippocampus (Zhang and Hernández, 2013) and LHb (unpublished data) has showed clearly the labeling in the lateral magnocellular AVP containing neurons. Hence, we consider that results from this study contribute to the unveiling of the complexity of the subcortical influence on the cortical physiological circuits.

### Technical considerations

#### Definition of “Axon” or “Axon-like” processes

We should clarify that the criteria used in this study for “axon” or “axon-like” processes were solely based on anatomical and immunohistochemical criteria as published in the literature (Sofroniew and Glasmann, 1981; van den Pol, 1982; Armstrong, 2004). These criteria include long distance projections with constant fiber diameter and ramification in the targeting regions. The presence of “string-of-pearls” appearance and strong AVP immunoreaction with granular appearance under confocal microscopy were the main criteria to assign axon-like fibers. The latter aspect is because most part of the dendrites lacks Golgi packaging organelles. We are fully aware that this is debatable and that ultrastructural features, such as the presence of axon-initial segment, remain to be determined by electron microscopy study.

#### Firing rate variations

All six AVP+ magnocellular neurons reported here increased their firing rate after hypertonic stimulus. However, their basal and post-stimulus firing rates were heterogeneous. Besides the expected individual and circadian time physiological variations, each surgery procedure could contribute to the basal firing rate since the AVP magnocells are particularly sensitive to blood volume variations and the close proximity between the penetration site and the central venous sinus on the brain surface caused frequent bleeding during the craniotomy.

#### Juxtacellular labeling and post-hoc anatomical study: advantages and limitations of this study

One of the main advantages of *in vivo* juxtacellular labeling for neuron reconstruction is that one can generally unequivocally connect the neurobiotin labeled processes (the main ones, at least), emitted from the labeled soma. The advantageous factors include the global fixation (not done on brain slices but done from integral animal without differential shrinkage between sections), the homogenous slicing, done by freely floating sections with a vibratome, and the resin treated relatively thick sections (70 μm) mounted on slides, which better conserves the 3D structure. With the help of a light microscope, at high magnifications, and a drawing tube, one can identify and clearly draw the trajectory and 3D coordinates of a given labeled process, especially its endings in X, Y, and Z planes (top vs. bottom). The accompanying anatomical structures, such as blood vessels, ventricles, prominent white matters, were also drawn on paper to help the *post-hoc* matching process.

There is always background “labeling” coming from endogenous biotin, mainly inside the mitochondria, which reacts with streptavidin or AB complex. However, at high magnification one can easily distinguish the endogenous background from labeling, since the endogenous patterns consisted in dots, contrasting with the exogenous tube-like continuous labeling pattern.

There were indeed processes labeled from non-labeled somata, the process indicated by a pink arrow in Figure 7C1a is an example. It was apparently a dendritic segment coming from an unlabeled soma. The tracer passed from the MM15 to this segment through a conjunction where a labeled axon from MM15 contacted the unlabeled cell's dendrite. This phenomenon has been reported in the literature and the presence of gap-junctions in this neuronal population had been suggested (Andrew et al., 1981).

In this study we did not use the neurohypophysis labeling as an inclusion criterion since in most of cases, this part of tissue was lost during the processing steps. The neurobiotin-axonal filling/visualization was incomplete in all cases. The time-lapse between labeling and perfusion/fixation, metabolism of the exogenous neurobiotin, and the possible presence of myelinization, among other factors, may contribute to this failure. As we reported in Figure 1, our success rate for this study was rather low (4%). Among other factors that hampered the experimental effectiveness, we could mention (1) the small size of the PVN; (2) the individual coordinate differences; (3) the high presence of pulsatile blood vessels (3.3 times) and longer lumen perimeters (3.6 times) more than in control regions of ventrolateral hypothalamus (van den Pol, 1982) and (4) the very low spontaneous firing rates (< 0.1 Hz) of AVP-magnocells under basal conditions in urethane anesthetized rats.

As we stated in the method section, most of the sections were processed and mounted—demounted 2–3 times, for different procedures. Some of them were air-dried and mounted in permount medium (detergent treated) and others underwent freeze-thaw procedure (non-detergent treated) and dehydrated with ethanol, propilene oxide and further contrasted with osmium for future electron miscroscopy studies. The permanently mounted sections were used for camera lucida reconstruction. In most cases, there were processes that were difficult to connect between sections. Among the possible factors for this limitation we would like to mention (1) the Z-collapse was variable for differently treated sections; (2) the fractures due to freeze thaw procedure and dehydration and (3) the high background in no detergent AB reaction. All these factors made the conections of distant axons/terminals not always feasible.

However, these results, in congruency with several previous reports in the literature, provided unequivocal evidence that the magnocells have an uncommon feature of possessing multiple axon-like processes emanating from the soma or a proximal dendrite. Furthermore, the long-range non-neurohypophyseal projections are more common than an “occasional” phenomenon as previously thought.

### Conflict of interest statement

The authors declare that the research was conducted in the absence of any commercial or financial relationships that could be construed as a potential conflict of interest.
